# Genomic Insight of *Alicyclobacillus mali* FL18 Isolated From an Arsenic-Rich Hot Spring

**DOI:** 10.3389/fmicb.2021.639697

**Published:** 2021-04-08

**Authors:** Martina Aulitto, Giovanni Gallo, Rosanna Puopolo, Angela Mormone, Danila Limauro, Patrizia Contursi, Monica Piochi, Simonetta Bartolucci, Gabriella Fiorentino

**Affiliations:** ^1^Dipartimento di Biologia, Università degli Studi di Napoli Federico II, Naples, Italy; ^2^Biological Systems and Engineering Division, Lawrence Berkeley National Laboratory, Berkeley, CA, United States; ^3^Institute of Polymers, Composites and Biomaterials (IPCB), Consiglio Nazionale delle Ricerche CNR, Pozzuoli, Italy; ^4^Istituto Nazionale di Geofisica e Vulcanologia, Sezione di Napoli Osservatorio Vesuviano, Naples, Italy

**Keywords:** geothermal environment, thermophilic microorganism, toxic metals, genomic sequencing and annotation, arsenic resistance system, bioremediation

## Abstract

Extreme environments are excellent places to find microorganisms capable of tolerating extreme temperature, pH, salinity pressure, and elevated concentration of heavy metals and other toxic compounds. In the last decades, extremophilic microorganisms have been extensively studied since they can be applied in several fields of biotechnology along with their enzymes. In this context, the characterization of heavy metal resistance determinants in thermophilic microorganisms is the starting point for the development of new biosystems and bioprocesses for environmental monitoring and remediation. This work focuses on the isolation and the genomic exploration of a new arsenic-tolerant microorganism, classified as *Alicyclobacillus mali* FL18. The bacterium was isolated from a hot mud pool of the solfataric terrains in Pisciarelli, a well-known hydrothermally active zone of the Campi Flegrei volcano near Naples in Italy. *A. mali* FL18 showed a good tolerance to arsenite (MIC value of 41 mM), as well as to other metals such as nickel (MIC 30 mM), cobalt, and mercury (MIC 3 mM and 17 μM, respectively). Signatures of arsenic resistance genes (one arsenate reductase, one arsenite methyltransferase, and several arsenite exporters) were found interspersed in the genome as well as several multidrug resistance efflux transporters that could be involved in the export of drugs and heavy metal ions. Moreover, the strain showed a high resistance to bacitracin and ciprofloxacin, suggesting that the extreme environment has positively selected multiple resistances to different toxic compounds. This work provides, for the first time, insights into the heavy metal tolerance and antibiotic susceptibility of an *Alicyclobacillus* strain and highlights its putative molecular determinants.

## Introduction

Arsenic is a widespread toxic metalloid component of the Earth crust and present in many geothermal environments (Nagy et al., 2014). It is also released into the environment by the consumption of arsenic-containing products such as insecticides, pesticides, and chemotherapeutic drugs (Bhowmick et al., 2018). Arsenic exists in two oxidation states, As(III) and As(V), and in inorganic and/or organo-metalloid forms (Qin et al., 2006). Due to its redox-active nature, arsenic is highly toxic as it may interfere in several biochemical reactions, by binding thiol groups of proteins or by substituting phosphate in oxidative phosphorylation (Nagy et al., 2014). Since arsenic causes numerous human diseases, it has been ranked by the World Health Organization among the top 10 chemicals that threaten public health and a class-I human carcinogen on the list of the IARC (International Agency for Research on Cancer, 2002; Mandal and Suzuki, 2002).

Geothermal environments are considered extreme habitats, not compatible with human life, because they combine different hostile conditions such as high temperature, salinity, acidity, and relevant metal concentrations; indeed, in acidic environments, the metal solubility is higher than in neutrophilic environments (Norambuena, 2020) and the levels of total arsenic can be up to 50 mg/l (Ballantyne and Moore, 1988). Such peculiar niches also represent exceptional reservoirs of poly-extremophiles, i.e., microbes adapted to live in the presence of more than one extreme condition (Saxena et al., 2017; Gallo et al., 2018; Schmid et al., 2020). Extremophilic microorganisms have attracted scientists for many reasons: they teach us about the origin and the limits of life (Bartolucci et al., 2013; Fusco et al., 2020); the use of their proteins/enzymes has boosted significant industrial and technological advances (Aulitto et al., 2018, 2019b; Fiorentino et al., 2020; Nayak et al., 2020); the elucidation of their molecular adaptations to survive in harsh conditions has revealed unique metabolic pathways and highlighted their potential as robust chassis for metabolic engineering (Fiorentino et al., 2003; Qin et al., 2006; Pothier et al., 2018). Application of extremophiles can further improve sustainability of biotechnological processes that can run more efficiently under harsh conditions (Krüger et al., 2018).

The exploration of the microbial diversity, the assessment of arsenic tolerance in volcanic water systems, and the microbial contribution to arsenic speciation are emerging fascinating fields covering culture-dependent and culture-independent approaches (Langner et al., 2001; Cuebas et al., 2011; Hug et al., 2014); in this regard, -omics techniques, such as metagenomics, genome sequencing, transcriptome and proteome analyses, and quantitative arsenic speciation data boost information on the complex relationship between chemical speciation and microbial metabolism and on the elucidation of the molecular mechanisms of arsenic tolerance in extreme environments (Langner et al., 2001; Wilkin et al., 2003; Aiuppa et al., 2006; Farnfield et al., 2012).

Such knowledge has set the basis to exploit microbial arsenic bioprocesses for the development of eco-sustainable approaches to deal with heavy metal pollution as well as to set up protein-based or whole-cell biosensors for the detection of heavy metals (Gomes et al., 2016; Politi et al., 2016; Krüger et al., 2018; Norambuena, 2020; Puopolo et al., 2021).

Microbes employ a variety of strategies to detoxify arsenic that include biochemical transformation (e.g., redox processes or methylation), extracellular precipitation, intracellular sequestration, and active extrusion from the cells (Páez-Espino et al., 2009).

The arsenic resistance genes are usually organized in *ars* operons, which include either three (*arsRBC*) or five (*arsRDABC*) genes: *arsR*, coding for As(III)-responsive transcriptional repressor controlling expression of *ars* genes; *arsC* encoding an arsenate reductase, which confers resistance to arsenate by converting arsenate into arsenite (Messens et al., 2002; Del Giudice et al., 2013); and *arsB* encoding an As(III) efflux protein. Acr3 is an unrelated As(III) antiporter (around 20–40% sequence similarity) that can be found as an alternative to ArsB (Yang et al., 2012), *arsA* encodes an As(III) stimulated ATPAse (Carlin et al., 1995), and *arsD* is a metallochaperone that favors arsenite transfer to ArsAB (Lin et al., 2006). The *arsRBC* operon is present in several microbes, among them *Staphylococcus aureus* plasmid pI258 (Ji and Silver, 1992), whereas *arsRDABC* has been characterized in *Escherichia coli* plasmid R773 and in other microorganisms (Saltikov and Olson, 2002). Many microbes possess an additional gene, *arsM*, coding for an arsenite methyltransferase that catalyzes the conversion of inorganic arsenic into mono-, di-, and tri-methylated products (Huang et al., 2018; Mestrot et al., 2020).

Among bacterial and archaeal extremophiles, enzymes for arsenic redox transformations and resistance systems have been characterized; for example, *Thermus* species use arsenate as the final acceptor of oxidative phosphorylation (Gihring and Banfield, 2001) and *T. thermophilus* HB27 has an arsenic resistance system composed of an arsenate reductase, an ArsR/SmtB transcriptional regulator, and an As(III) efflux transporter (Antonucci et al., 2017; Gallo et al., 2019). The extremely resistant archaeon *Ferroplasma acidarmanus* co-transcribes and expresses *arsB* and *arsR* in response to As(III), by compensating the lack of *arsC* in the genome (Gihring et al., 2003); other phylogenetically distinct archaea including *Pyrobaculum calidifontis*, *Sulfolobus tokodaii*, and *Aeropyrum pernix* are As(III)-oxidizing microbes that use a membrane arsenite oxidase encoded by widespread *aio* clusters (Mikael Sehlin and Börje Lindström, 1992; Shi et al., 2020).

A considerable number of microbial genomes has highlighted even greater complexity of the arsenic resistance determinants; for example, resistance genes can be often found scattered in the genome, duplicated or present in multiple copies; it has been suggested that redundancy of gene configurations in *ars* gene clusters has arisen from horizontal gene transfer and is responsible for the increased arsenic tolerance (Ordóñez et al., 2005; Li and Krumholz, 2007; Páez-Espino et al., 2015). In extremophilic microorganisms, genomic islands encoding for multiple resistances would have been acquired by the same microbe, conferring it capabilities to face multiple metals (Jackson et al., 2005; Norambuena, 2020).

The present study aimed to deepen knowledge on the genetic determinants of heavy metal tolerance, particularly arsenic, in microbes thriving in extreme environments; in this context, we chose as isolation site Pisciarelli, a hot spring located in the volcanic area of Campi Flegrei in Italy. It displays an impressive and powerful hydrothermal activity with a puzzling of various hot and acidic environments (Baquero et al., 2020) induced by fumaroles at temperature up to ∼110°C, vigorous boiling pools and diffuse soil degassing. From year 2006, these conditions have been continuously changing and increasing, due to the effect of the endogenous engine and the meteoric agents (Valentino and Stanzione, 2003; Chiodini et al., 2010; Piochi et al., 2015, 2019; Cardellini et al., 2017).

Therefore, we describe herein the isolation of a novel thermoacidophilic bacterium, *Alicyclobacillus mali* FL18, from a hot mud pool at Pisciarelli and its genomic and physiological characterization.

## Materials and Methods

### Chemicals

The metal salts used in this work were purchased by Sigma-Aldrich and they are as follows: Sodium (meta) arsenite (NaAsO_2_), Sodium arsenate dibasic heptahydrate (Na_2_HAsO_4_ ⋅ 7H_2_O), Cadmium chloride (CdCl_2_), Cobalt chloride (CoCl_3_), Copper chloride (CuCl_2_), Mercury chloride (HgCl_2_), and Nickel chloride (NiCl_2_). The antibiotics used were also purchased by Sigma-Aldrich and are ampicillin (CAS Number: 7177-48-2), bacitracin (CAS Number: 1405-87-4), chloramphenicol (CAS Number: 56-75-7), ciprofloxacin (CAS Number: 85721-33-1), erythromycin (CAS Number: 114-07-8), kanamycin sulfate (CAS Number: 70560-51-9), streptomycin (CAS Number: 3810-74-0), tetracycline (CAS Number: 60-54-8), and vancomycin (CAS Number: 1404-93-9).

### Study of the Area and Sampling

**FIGURE 1:**
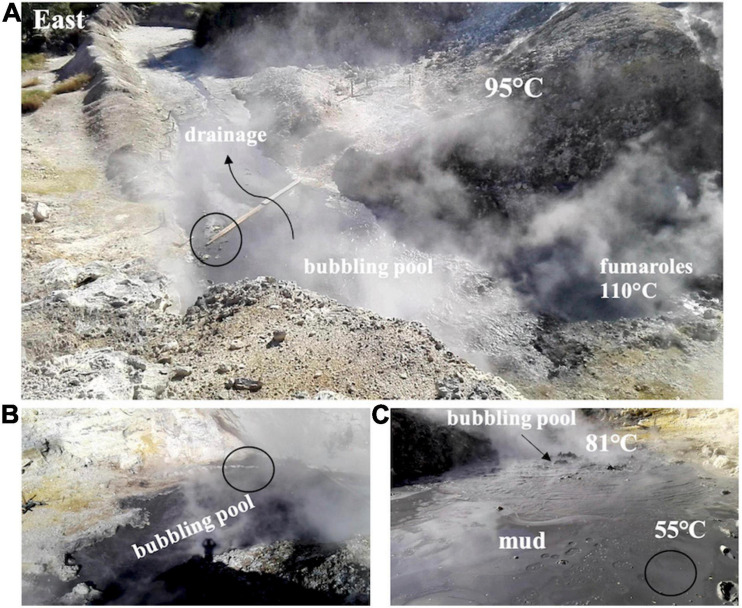
The Pisciarelli site, the sample mud pool, and the sample area (white circle). **(A)** The hydrothermal setting with the fumaroles, the main boiling pool, the outflow water drainage zone, and the surrounding diffuse degassing area. **(B)** Southern view of the bubbling pool with the boiling points. **(C)** The sampled muddy area at the periphery of the mud pool.

The samples were collected in September 2018 at Pisciarelli (Figure 1A), an active portion of the wider volcanic field of Campi Flegrei close to Naples in Italy. The area located on the northeastern slope of the Solfatara cone degasses sulfurous water vapors and over 300 g/m^2^ per day of CO_2_ (Chiodini et al., 2010; Piochi et al., 2015; Cardellini et al., 2017). This hot spring is characterized by the presence of a main bubbling mud pool with temperatures up to 80–85°C and marginal water-poorer portions with lower temperature (Piochi et al., 2019). The levels of arsenic are included in the 10–15 ppm range in the mud and in the 39–2,000 μg/L range in waters reaching up to 6,000 μg/L in nearby zones (Valentino and Stanzione, 2003). The samples were aseptically collected close to the marginal water-poorer portion (Figures 1B,C) where the temperature and the pH were ∼55°C and 5.0, respectively.

### Cultivation and Isolation of Growing Bacteria

To select thermophilic and acidophilic bacteria, the collected samples were enriched in 20 ml of modified Luria-Bertani (LB) (adjusted to pH 5 through addition of HCl) and incubated for 24 h at 55°C with a shaking rate of 180 rpm, in a MaxQTM 4000 Benchtop Orbital Shaker (Thermo Scientific). The enriched sample was plated on LB agar pH 5.0 at 55°C and grown for 48 h. Single colonies were isolated through repeated streak-plating and then the isolate was identified through 16S rRNA gene sequencing followed by data analysis through the EMBL database^1^. The sequence could be identified as belonging to a member of the genus *Alicyclobacillus*.

### Genomic DNA Isolation and Sequencing

Genomic DNA extraction from *Alicyclobacillus* spp. was performed using LETS buffer (0.1 M LiCl, 0.01 M EDTA, 0.01 M Tris–HCl, pH 7.4, and 0.2% SDS) and phenol extraction as already described (Aulitto et al., 2019a). Final yield and quality of DNA were determined spectrophotometrically using a Qubit Fluorometer (Invitrogen Co.). One hundred nanograms of genomic DNA was then used to prepare indexed libraries with Nextera DNA Flex Library Prep (Illumina) according to the manufacturer’s instructions. The library was quantified using Qubit fluorometer (Invitrogen Co.) and then sequenced on NextSeq550 platform (Illumina, San Diego, CA, United States) in a 2 × 75 paired-end format. Whole-genome sequencing was performed at Genomix4life s.r.l. (Salerno, Italy).

### Genome Assembly and Annotation

A total of 2,309,234 reads (average length, 75 bp) were assessed through FastQC v 0.11.5 (Brown et al., 2017). Different assemblers SPAdes v 3.13.0 (Bankevich et al., 2012), IDBA-UD v 1.1.3 (Peng et al., 2012), and MEGAHIT v 1.2.9 (Li et al., 2015) were used and the results compared using QUAST v 4.4 (Mikheenko et al., 2016). Furthermore, the assembly was evaluated for completeness and contamination by means of CheckM v 1.0.18 (Parks et al., 2015). For comparative genomics and gene analysis, a Rapid Annotations using Subsystems Technology (RAST) v 0.1.1 was used to perform the annotation (Aziz et al., 2008). This analysis was used to identify (i) arsenic resistance genes, (ii) heavy metal resistance genes, and (iii) antibiotic resistance genes, by performing BLASTp and HMM-based procedures included in RAST system. EggNOG (Evolutionary Genealogy of Genes: Non-supervised Orthologous Groups) database was employed to assign a functional annotation of the identified orthologous groups and to hamper the interpretation of subsequent results (Huerta-Cepas et al., 2019). The orthologous clusters were visualized using the online service OrthoVenn2^2^ (Xu et al., 2019).

### Comparative Genomic Analysis

To construct the phylogenetic tree, different sequences of *Alicyclobacillus* species available at National Center for Biotechnology Information (NCBI) genome sequence repository^3^ were compared using SpeciesTreeBuilder v.0.1.

The average nucleotide identity (ANI) was determined using the online ANI calculator based on an improved OrthoANIu algorithm^4^. *In silico* DNA–DNA hybridization (DDH) values were estimated using the Genome-to-Genome Distance Calculator (GGDC)^5^. ANI values of 95–96% and DDH value of 70% were used as a boundary for species delineation. A graphical genome view was generated employing CGView software (Grant and Stothard, 2008).

### *A. mali* FL18 Growth Conditions

In order to identify the optimal growth conditions of *A. mali* FL18, growth curves were followed in three different media: Luria Bertani medium (LB) pH 4.0, Yeast Starch Glucose medium (YSG) pH 4.0, and *Bacillus acidoterrestris* thermophilic medium (BAT, HiMedia Biolabs), pH 4.0 (Anjos et al., 2014). A frozen (−80°C) glycerol-stock of *A. mali* FL18 was incubated in 5 ml of each medium for 16 h at 55°C and then diluted at 0.1 OD_600 *nm*_ in fresh medium, and the growth measured shaking for 16 h at 55°C in a Synergy^TM^ HTX Multi-Mode Microplate Reader (BioTek, United States). Each experiment was performed in technical and biological triplicates.

### Heavy Metal Ion Tolerance

For the determination of Minimal Inhibitory Concentration (MIC) toward the heavy metal ions [As(V), As(III), Cd(II), Co(III), Cu(II), Hg(II), Ni(II)], exponentially growing cells were diluted to 0.1 OD_600 *nm*_ in 600 μl of BAT medium (pH 4.0) containing metal ion at concentrations ranging from 0.5 μM to 45 mM. The cultures were incubated at 55°C for 16 h and MIC values were determined as the lowest concentration of metals that completely inhibited the growth of *A. mali* FL18 adopting the procedure already described (Antonucci et al., 2017, 2018). The reported values are the average of three biological replicates.

### Determination of Arsenic Tolerance

To evaluate the effect of arsenate and arsenite on the bacterial growth, an overnight culture was diluted at 0.1 OD_600 *nm*_ in fresh BAT medium and grown in the presence of 20 mM As(III) and 6 mM As(V). The growth curves were recorded measuring the OD_600 *nm*_ for 16 h at 55°C under shaking in a Synergy^TM^ HTX Multi-Mode Microplate Reader (BioTek, United States). The generation time (*G*) was calculated as: *G* = *t*/*n*, where *t* is the time interval and *n* the number of generations (measured between 2 h and 3 h, in the exponential phase). All the experiments were repeated in triplicate.

### Antibiotic Susceptibility

MIC toward different antibiotics was determined using a procedure previously described (Puopolo et al., 2020). In detail, the bacterial culture was diluted up to 0.1 OD_600 *nm*_ in BAT medium, pH 4.0, supplemented with concentrations ranging from 0.5 to 1,000 μg/ml of ampicillin, bacitracin, chloramphenicol, ciprofloxacin, erythromycin, kanamycin, streptomycin, tetracycline, and vancomycin, and grown at 55°C for 16 h. MIC was evaluated by measuring OD_600 *nm*_ after incubation for 16 h in the presence of different concentrations of antibiotics. The reported values are the average of three biological replicates.

### Arsenic Biotransformation by *A. mali* FL18

A colorimetric assay based on the formation of precipitates upon reaction of AgNO_3_ with arsenic was set up to address biotransformation of arsenate into arsenite, and *vice versa*, by *A. mali* FL18 (Simeonova et al., 2004). A single colony was inoculated in BAT medium, pH 4.0, at 55°C for 6 h, and then it was diluted to 0.1 OD_600 *nm*_ in 1 ml of BAT medium containing 5 mM Na_2_HAsO_4_ and 20 mM NaAsO_2_; an arsenic-free control culture (CN) was also grown. The cultures were incubated at 55°C for 16 h, and cells were harvested by centrifugation at 6,000 rpm for 10 min (Eppendorf Centrifuge 5804 R); the supernatants and pellets were flooded with 100 μl of 0.1 M AgNO_3_. The color of the precipitate on each sample was compared to two reference color scales each obtained by mixing defined ratios of As(V) and As(III) (100:1, 75:25, 50:50, 25:75, and 1:100) at final concentrations of total arsenic of 5 mM or 20 mM. This analysis gives a qualitative indication of arsenic transformation. All the experiments were repeated in triplicate.

## Results and Discussion

### Site Description, Sampling, and Identification of a New Strain of *Alicyclobacillus*

This study focuses on the isolation and identification of a heavy metal tolerant bacterium from a well-known solfataric environment, located in the volcanic area of Campi Flegrei close to Naples, Italy. Hot springs are unique niches of biodiversity, and microbes living under intense selective pressure are found to be part of consortia sharing temperature, pH, *in situ* chemistry, and biogeography (Portillo et al., 2009). In these communities, microorganisms have evolved strategies to thrive in harsh conditions by converting chemicals and organic matter into cellular energy (Aulitto et al., 2020). In the biogeochemical cycle of arsenic, the microbiome plays a key role contributing to metal mobilization as well as to shape the structure of the ecosystem in which it thrives (Langner et al., 2001; Donahoe-Christiansen et al., 2004).

Pisciarelli was selected as the ideal isolation site because, as known for other volcanic areas, it is characterized by aggressive sulfurous effluxes and significant concentration of certain elements such as arsenic and mercury over 50 ppm (Valentino and Stanzione, 2003; Piochi et al., 2019). It has been shown that sampling in acid-sulfidic geothermal systems can give insight into the effects of microbial communities on arsenic and sulfur biogeochemical cycles and increase the knowledge on the relationship between arsenic resistance and sulfur cycling (Hug et al., 2014).

Table 1 summarizes main physical–chemical features of the Pisciarelli site. In particular, regarding sulfur forms, H_2_S is the main gas species, whereas in the water and mud/soil, the stable form is SO_4_^2–^. Pyrite (FeS_2_) and native sulfur (S_8_) occur within the mineral association of mud and soil; total S is between ca. 11 and 30% in mud, and SO_4_^2–^ is between ca. 1,300 and 7,100 mg/L in water (Table 1). Regarding arsenic forms, As(V) oxy-anions (HAsO_4_^2–^, AsO_4_^3–^) are predominant, in agreement with the highly oxidant conditions and the geothermal outgas (Aiuppa et al., 2006). Moreover, dimeric As-S complexes (H_2_As_2_S_4_) are the major As aqueous species at equilibrium under sulfur-rich conditions.

**TABLE 1:** Physico-chemical conditions at the Pisciarelli site.

		Solid	Water	Fumarole
Temperature (°C)	94.3		83–96^3^	116
pH	4.8		1.4–4.9^3^	n.d.
TDS			2060–9100^3^	
Minerals^1^	–	Alunite, sanidine, pyrite, sulfur, hydroBiotite/illite, amorphous	Illite/montmorillonite, ammonium K sulfate	–
Main sulfur state	SO_4_^2–^	SO_4_^2–^, S^0^, sulfides (mostly S^–1^)	SO_4_^2–^	H_2_S
H_2_S flux	0.091 tons/day^2^	–	–	0.849 tons/day^2,3^
SO_2_ flux	n.d.	–	–	0.0014 tons/day^2^
CO_2_ flux	48.3 tons/day^2^	–	–	231–300 tons/day^2,3^
H_2_ flux	0.0033 tons/day^2^	–	–	0.0227 tons/day^2^
H_2_O content	–	–	–	809,000–870,000 μmol/mol^4^
CO_2_ content	–	–	–	115,000–188,000 μmol/mol^4^
H_2_S content	–	–	–	517–696 μmol/mol^4^
H_2_ content	–	–	–	137–294 μmol/mol^4^
CH_4_ content	–	–	–	9.1–19 μmol/mol^4^
Al (as Al_2_O_3_ in solid)	–	10–11.85 wt%^5^	19–66 mg/L^6^	–
Na (as Na_2_O in solid)	–	0.24–0.42 wt%^5^	7.7–122 mg/L^6^	–
Fe (as Fe_2_O_3_ in solid)		2.3–3.1 wt%^5^	34–161 mg/L^6^	
Cl	–	n.a.	6–83 mg/L^6^	–
NH_4_^+^	–	n.a.	501–1026 mg/L^6^	–
HCO_3_^2–^	–	–	n.d.	–
SO_4_^2–^ (as S in solid)	–	10.62–31.12 wt%^5^	1319–7043 mg/L^6^	–
F	–	n.a.	0.1–30 mg/L^6^	n.d.
C	–	0.14–0.21 wt%^5^	–	–
As	–	9.5–12.9 ppm^5^	39–1880 μg/L^6^	–
Cd	–	<0.1 ppm^5^	n.a.	–
Cu	–	7.9–10.6 ppm^5^	n.a.	–
Co	–	3.4–5.7 ppm^5^	n.a.	–
Ni	–	2.6–3.1 ppm^5^	n.a.	–
Hg	–	24.98–41.95 ppm^5^	40–232 μg/L^6^	–
Pb	–	14.8–20.1 ppm^5^	5.7–29.1 mg/L^6^	

*^1^Detected by X-ray diffraction and energy dispersive microanalytical system-equipped electron microscope (Istituto Nazionale di Geofisica e Vulcanologia, Osservatorio Vesuviano labs in Naples, Italy) on mud (solid) and dried water separated from investigated sample. ^2^Average flux values in 2013 by Aiuppa et al. (2013). ^3^Flux values in 2018 by Tamburello et al. (2019). ^4^From Caliro et al. (2007). ^5^From Piochi et al. (2019). ^6^From Valentino et al. (1999) and Valentino and Stanzione (2004). n.d., not detected; n.a., not analysed. Values for element are content in terrain/mud matrices and content dissolved in waters.*

The occurrence of heavy metals is also reflected by the presence of mineral precipitates, i.e., realgar (As_4_S_4_) and pyrite (FeS_2_) coexisting with native sulfur, sulfates, and sulfides and amorphous silica found around the main fumaroles and within the mud pool (Piochi et al., 2015, 2019). Other metals found in the solfataric terrains of Pisciarelli are nickel, copper, and cobalt, at <20 ppm, whereas cadmium was not detected (Piochi et al., 2019).

At the time of sampling, the marginal water pool temperature was ∼55°C and the pH was ∼5 (Figure 1). To mimic the sampling site, the collected samples were initially grown in LB at pH 5 at 55°C. After serial dilutions and repeated streaking procedures on solid medium, a single colony was isolated and identified through 16S rRNA sequencing. This analysis allowed the identification of a member of the *Alicyclobacillus* genus. First representatives of the *Alicyclobacillus* genus were initially isolated from the hot springs of the Yellowstone National Park and the Hawaii Volcano National Park in the United States and from the hydrothermal waters of Solfatara (Darland and Brock, 1971; De Rosa et al., 1971); these sites share similar acidic sulfate conditions (Aiuppa et al., 2006). The genus has been named *Alicyclobacillus* since 1992 to include several isolates previously wrongly attributed to the *Bacillus* genus (Wisotzkey et al., 1992). *Alicyclobacillus* are strictly aerobic thermo-acidophilic microorganisms that produce ω-alicyclic fatty acids as major cellular fatty acids (Hippchen et al., 1981; Poralla and König, 1983) and grow optimally at temperatures between 28 and 65°C (Baumgart, 2003; Ciuffreda et al., 2015). After their discovery, a high number of enzymes have been characterized from those microbes that helped to define the molecular determinants of thermostability (Bartolucci et al., 1997; Atalah et al., 2019). To date, 19 species belonging to the genus *Alicyclobacillus* have been identified (Ying et al., 2010; Smit et al., 2011; Pornpukdeewattana et al., 2020) and are grouped into three categories depending on their growth temperature ranges: (1) in the 45–70°C range, with a temperature optimum of 65°C; (2) in the 20–65°C range, with an optimum set at 40–55°C; and (3) the range of 4–55°C with optimum growth temperatures from 35°C to 42°C (da Costa et al., 2015).

### Genome Assembly and Functional Annotations

**FIGURE 2:**
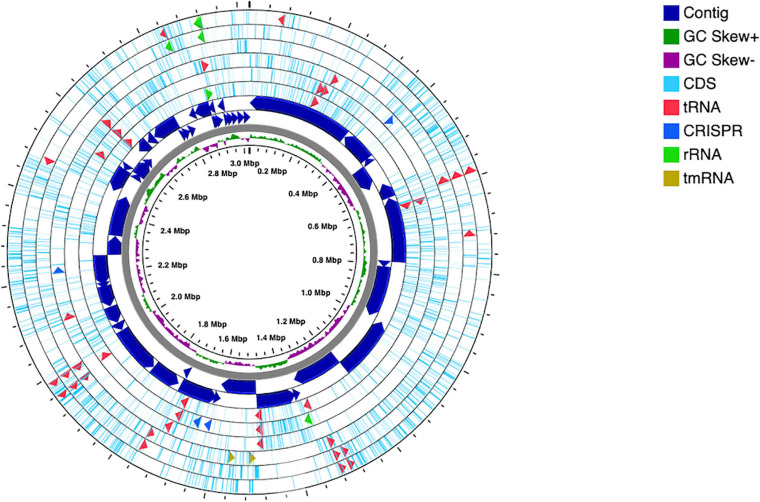
Circular map of *Alicyclobacillus mali* FL18 genome performed using CGview Server. From the center to outside: genome size, with a ring showing the GC skews (positive values in green and the negatives in purple); the contigs, represented as dark blue arrows in a double ring to indicate the strands plus and minus; the CDSs are reported in light blue, together with tRNA (red), rRNA (green), tmRNA (dark red), and CRISPR (blue).

The genome was successfully sequenced and the statistics are summarized in Table 2. Different assembler tools were tested, and the best assembly was obtained using MEGAHIT, which comprises 48 contigs (all of them ≥1,000 bp) with a total length of ∼3 Mbp, N50 value of 128,277 bp, and an average GC content of 61.5%. To assess the genome quality, the assembly was evaluated for completeness (99.61%) and contamination (0.0%) using CheckM. Annotation of the genes in the *Alicyclobacillus* genome was carried out using RAST server and eggNOG databases. The analysis obtained from RAST revealed the presence of 3127 coding sequences, 64 RNAs, of which 62 tRNA and 2 rRNA (LSU and SSU). Moreover, the presence of 4 CRISPR arrays with 91 CRISPR spacers and 99 CRISPR repeats can be correlated to the existence of the adaptive immunity system CRISPR-Cas (Figure 2). The high number of CRISPR spacers can be explained by the complexity of the ecosystems from which the microorganism was isolated and by the targeting of the bacterial host from many phage (Guerrero et al., 2020).

**TABLE 2:** Genome statistics of *Alicyclobacillus mali* FL18.

Genome	*Alicyclobacillus mali* FL18
Domain	Bacteria
Size (bp)	3,024,307
GC content (%)	61.5
N50 (bp)	124,285
L50	9
Number of contigs (with PEGs)	48
Number of subsystems	271
Number of coding sequences	3127
Number of RNAs	64

**FIGURE 3:**
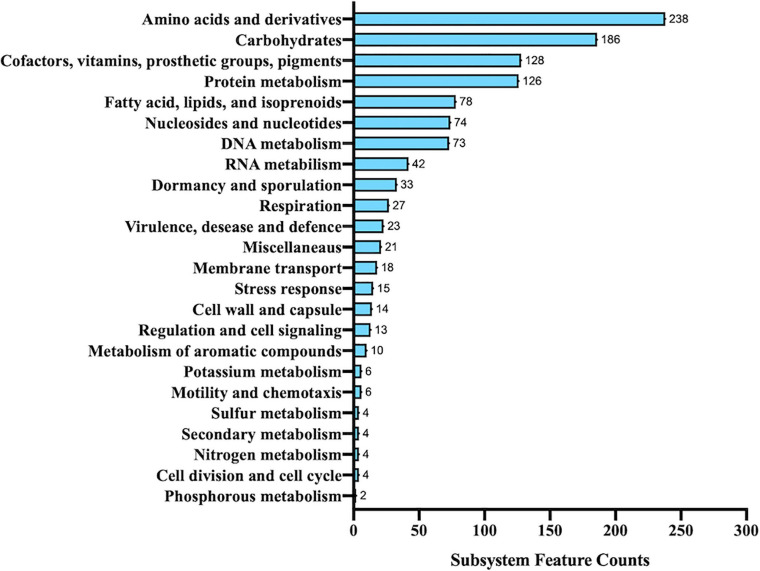
Functional annotation of *Alicyclobacillus* isolate using RAST server.

Interestingly, only 26% of the predicted genes was recognized by RAST and classified in 271 subsystems. The distribution of different functional groups showed a predominance of genes involved in general processes related to amino acid and carbohydrate metabolism, but several genes were also found to be involved in the biosynthesis of cofactors, vitamins, prosthetic groups, pigments, and protein metabolism (Figure 3). Interestingly, 78 genes were included in the subsystem of fatty acids, lipids, and isoprenoids according to the presence of a peculiar fatty acid metabolism in *Alicyclobacillus*; in particular, isoprene molecules have many physiological roles and have found several applications as fragrances, essential oils, and, most recently, biofuels (Phulara et al., 2016).

To expand the functional annotation of the isolated strain, we established orthology relationships between the sequenced genome of the *Alicyclobacillus* isolate and other annotated genomes, using the last version of the eggNOG (Huerta-Cepas et al., 2019). For this purpose, 2,507 genes were predicted and functionally annotated using KEGG pathways, Gene Ontology (GO) terms, and Clusters of Orthologous Groups of proteins (COGs), the latter listed in Table 3. Considering all the protein encoding genes, we found that 80.97% matched with COG functional categories and the remaining 19.03% persisted as unclassified; moreover, 42.68% of annotated proteins were clustered within the functions associated to specific metabolisms; in particular, many of them were found to be associated with amino acid transport and metabolism (E), energy production and conversion (C), and carbohydrate transport and metabolism (G). This result agrees with the annotation data obtained from RAST subsystems described above.

**TABLE 3:** Clusters of Orthologous Groups of proteins (COG) classification of the annotated genes in *A. mali* FL18.

	COG description	COG	Value	% of total
Cellular processes and signaling	Cell cycle control, cell division, chromosome partitioning	D	52	2.07
	Cell wall/membrane/envelope biogenesis	M	124	4.95
	Cell motility	N	59	2.35
	Post-translational modification, protein turnover, and chaperones	O	73	2.91
	Signal transduction mechanisms	T	65	2.59
	Intracellular trafficking, secretion, and vesicular transport	U	28	1.12
	Defense mechanisms	V	34	1.36
Information storage and processing	Translation, ribosomal structure, and biogenesis	J	160	6.38
	Transcription	K	197	7.86
	RNA processing and modification	A	1	0.04
	Replication, recombination, and repair	L	167	6.66
Metabolism	Energy production and conversion	C	161	6.42
	Amino acid transport and metabolism	E	251	10.01
	Nucleotide transport and metabolism	F	73	2.91
	Carbohydrate transport and metabolism	G	168	6.70
	Coenzyme transport and metabolism	H	123	4.91
	Lipid transport and metabolism	I	129	5.15
	Inorganic ion transport and metabolism	P	131	5.23
	Secondary metabolites biosynthesis, transport, and catabolism	Q	34	1.36
Poor char	Function unknown	S	477	19.03
			2507	100.00

### Taxonomic Affiliation and Phylogenetic Analysis

**FIGURE 4:**
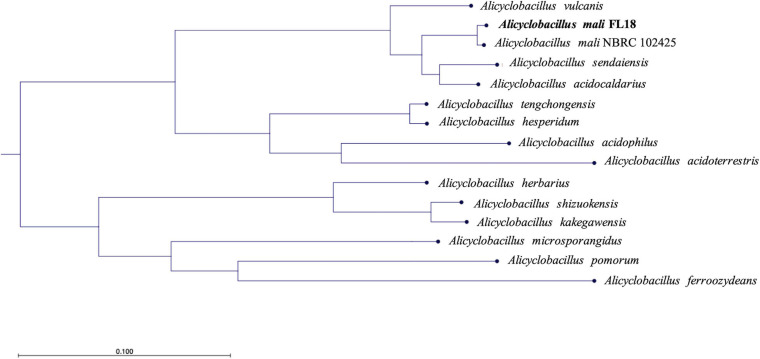
Phylogenetic tree based on the genome comparison of *A. mali* FL18 with reference genomes publicly available at NCBI.

To shed light on the taxonomy of the new isolated *Alicyclobacillus*, we resolved to perform a phylogenetic analysis. In fact, the analysis of 16S rRNA was not straightforward to unambiguously identify the species, because, as reported in the literature, 16S rRNA sequences are very similar among members of the genus *Alicyclobacillus* (Connor et al., 2005; Goto et al., 2008). In detail, a phylogenetic tree was built using a COG approach, which is based on the analysis of universally conserved genes defined by COG families (Figure 4 and Supplementary Table 1). This procedure is based on the notion that if small groups of proteins (at least three) from different genomes are similar to each other, they can belong to an orthologous family (Livingstone et al., 2018). By applying this method, a subset of closely related genomes was imported using RefSeq (NCBI), and the relatedness was calculated from a multiple sequence alignment (MSA) for each COG family. Afterward, the poorly aligned sections were trimmed and concatenated to reconstruct the phylogenetic tree (Figure 4). The isolate resulted to be closely related to *A. mali* NBRC 102425 deposited on NCBI (Accession number: NZ_BCSG01000001). Given the high evolutionary relationship between the two microbes, we turned out to assess whether the *Alicyclobacillus* isolate and *A. mali* NBRC 102425 belonged to the same species; for this reason, we calculated the average nucleotide identity (ANI) of the two whole genomes. ANI is built as an alignment-based search that gives a similarity index between two genomes; a cutoff score of >95% is indicative of members belonging to the same species (Jain et al., 2018). Indeed, the ANI value resulted to be 98.91% (Supplementary Table 2), strongly suggesting that the *Alicyclobacillus* isolate belonged to the species *mali*. To confirm the result, the Genome-to-Genome Distance Calculator (GGDC), was employed (Auch et al., 2010). Also, in this case, the DDH value was calculated using as genome comparison *A. mali* NBRC 102425. From the data obtained using a generalized linear model (GLM), the DDH value was 89.70% indicating that they belong to the same species (Supplementary Table 2). On the other hand, the value calculated to estimate the probability that the two bacteria belonged to the same sub-species was 64.81%, lower than the threshold (Supplementary Table 2) (Meier-Kolthoff et al., 2013). Altogether, these results suggest that the isolate is a new strain of *A. mali*. Hereinafter, the isolated strain was named *A. mali* FL18 and the complete genome sequence was deposited at NCBI GenBank under accession number JADPKZ000000000.

### Comparative Genomics of *A. mali* Strains

**FIGURE 5:**
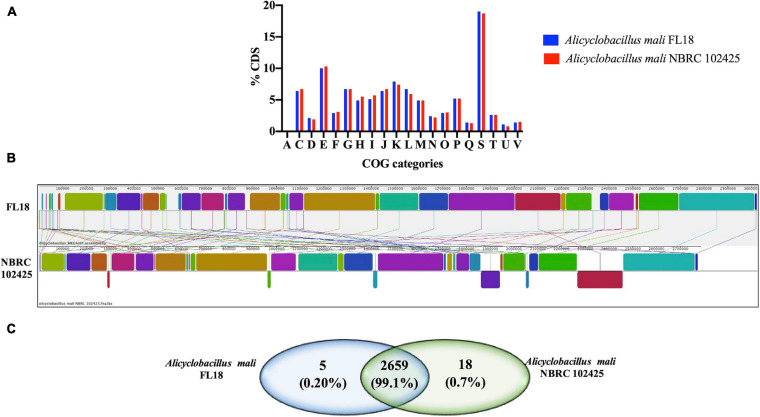
Genome comparison between *A. mali* FL18 and NBRC 102425. **(A)** COG comparison of *A. mali* FL18 (blue) and *A. mali* NBRC 102425 (red). **(B)** Mauve progressive alignment of FL18 and NBRC 102425. **(C)** Venn diagram showing the number of shared and unique proteins.

A comparative analysis was performed to gain insights into the similarities and differences between the two *A. mali* strains (FL18 and NBRC 102425). The genome sizes of *A. mali* FL18 and *A. mali* NBRC 102425 turned out to be slightly different, i.e., 3,024,307 and 2,786,970 bp, respectively, while the GC content was comparable (61.5 and 61.9%) (Supplementary Table 3). The genomes of both strains were annotated using RAST server and the predicted DNA coding sequences (CDS) were 3127 and 2815 for FL18 and NBRC102425 strains, respectively (see also above). Of these CDSs, 2386 (84.8%) and 2507 (80.2%) belonged to the COG families. In fact, Figure 5A shows how the distribution patterns resemble each other.

To evaluate the differences at sequence level between the strains in more detail, the bioinformatic tool Mauve was exploited (Darling et al., 2004). The graphical analysis obtained after nine steps of progressive alignments shows the presence of well conserved regions, but clearly randomly distributed (Figure 5B). This similarity between the two strains FL18 and NBRC 102425 was also confirmed at protein level using OrthoVenn, a web platform based on the comparison and analysis of whole-genome orthologous clusters (Xu et al., 2019). In this case, the analysis confirms that 99.1% of the proteins encoded from the two *mali* strains are shared (Figure 5C).

### Mining of the Arsenic Resistance Genes in *A. mali* FL18 Genome

**FIGURE 6:**
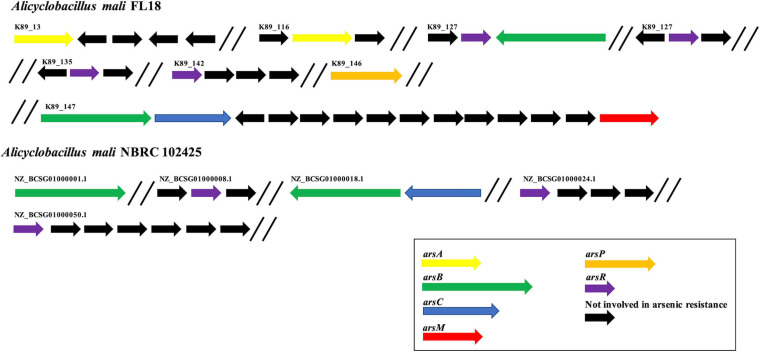
Organization of arsenic resistance genes. Comparison of arsenic resistance genes in *A. mali* FL18 **(Up)** and *A. mali* NBRC 102425 **(Down)**. In purple, *arsR* genes coding for arsenic responsive transcriptional regulator; in blue, *arsC* genes coding for arsenate reductase; in green, *arsB* genes coding for arsenite membrane transporter; in yellow, *arsA* genes coding for anion stimulated ATPase; in red, *arsM* genes coding for arsenite methyltransferase; in orange, *arsP* genes coding for organoarsenical permease; in black, genes that are not involved in arsenic resistance.

Considering the presence of high concentration of arsenic in the extreme environment of Pisciarelli (Piochi et al., 2015, 2019), we attempted to identify potential genes involved in arsenic resistance using RAST annotation server (Aziz et al., 2008). In this annotation, the central component for protein identification is FIGfams, a collection of protein families classified in sets of iso-functional homologs (Aziz et al., 2008). RAST annotation highlighted the presence of an arsenal of 11 arsenic resistance genes in *A. mali* FL18 genome, encoding for putative proteins related to arsenic resistance (Table 4). Of these putative genes, only two were clustered (one copy of *arsB* and one copy of *arsC*), whereas the majority of putative *ars* sequences were scattered on the genome (Figure 6). The arsenate reductase gene *arsC* encodes an arsenate reductase belonging to the family of thioredoxin-coupled arsenate reductases, already found in other thermophilic microorganisms (Messens et al., 2002; Del Giudice et al., 2013). Additionally, two hypothetical *arsB* genes and two *arsA* genes were also found that could functionally associate in ArsAB complexes to guarantee more efficient arsenite extrusion. Moreover, four genes putatively coding for ArsR, the arsenic resistance transcriptional regulator, were detected; these proteins are metal sensor DNA-binding proteins that act as repressors, by dissociating from DNA in the presence of metal ions, thereby allowing the expression of the metal resistance genes (Wu and Rosen, 1991). As already observed in other thermophilic microorganisms, the *ars* genes are interspersed in the genome and can be controlled by multiple ArsR transcription factors (Ordóñez et al., 2005; Antonucci et al., 2017).

**TABLE 4:** Annotation, strand, and length of the predicted arsenic resistance genes.

Function	Strand	Length
Transcriptional regulator, ArsR family	+	239
Transcriptional regulator, ArsR family	+	96
Transcriptional regulator, ArsR family	+	312
Transcriptional regulator, ArsR family	+	112
Arsenate reductase (EC 1.20.4.4) thioredoxin-coupled, LMWP family	+	140
ArsB, Arsenite/antimonite:H^+^ antiporter	+	430
ArsB, Arsenite/antimonite:H^+^ antiporter	−	399
ArsA, ATPase	+	384
ArsA, ATPase	+	365
ArsM, arsenite methyltransferase SAM-dependent	+	278
ArsP, arsenite permease	+	167

Regarding resistance to organoarsenicals, a gene encoding for an arsenite methyltransferase (*arsM*) and a hypothetical methylarsenite efflux permease (*arsP*) were found; their encoded proteins could contribute to transform and extrude methylated forms of arsenite (Wang et al., 2015). The increasing number of sequenced genomes and comparative studies has elucidated the distribution of arsenic determinants in several species, revealing that genes coding for arsenic-related processes are phylogenetically and ecologically widespread in bacteria and archaea, either mesophiles or thermophiles (Andres and Bertin, 2016). In our case study, the repertoire of arsenic resistance genes of *A. mali* FL18 was compared to that of *A. mali* NBRC 102425, and the analysis showed that our isolate has a more complex resistance system (Figure 6). Therefore, it is expected that it could be more tolerant to arsenic than the NBRC 102425 strain and represent a model system to unravel sophisticated arsenic resistance mechanisms.

Furthermore, to analyze the correlation between sulfur metabolism and arsenic tolerance, we looked for the presence in *A. mali* FL18 of genes responsible for reduction and fixation of sulfur into biomolecules. According to the genomic contest, neither sulfur, nor sulfide or sulfite oxygenases, nor oxidases or dehydrogenases were found, whereas genes involved in the sulfur reduction and fixation were discovered (NCBI Accession Number WP_195867833.1_2008; WP_195867500.1_1409; WP_067850267.1_2663; WP_195867655.1_935; and WP_195867654.1_934). This analysis strongly suggests that *A. mali* FL18 is not a sulfur-oxidizing bacterium; nevertheless, the correlation between arsenic and sulfur biogeochemistry in Pisciarelli hot spring could depend on the complexity of yet undiscovered microbial communities and their intricate contribution to the biogeochemical cycle of arsenic, as also demonstrated by the presence of several minerals like FeS_2_ and As_4_S_4_.

### *In silico* Analysis of the Heavy Metal and Antibiotic Resistance Determinants in *A. mali* FL18 Genome

**FIGURE 7:**
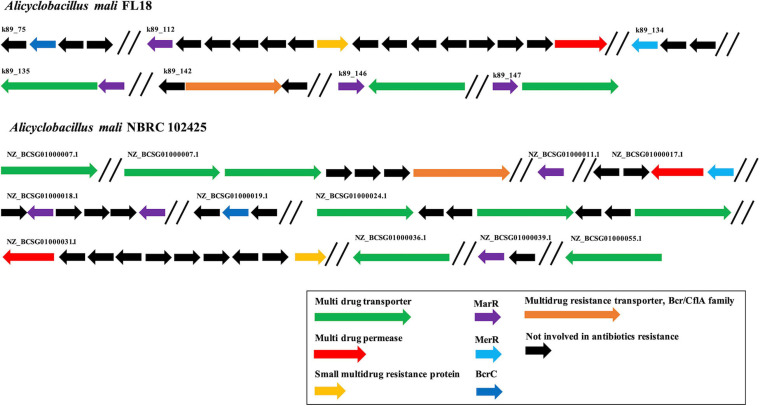
Organization of multi-drug resistance genes. Comparison of antibiotic resistance genes in *A. mali* FL18 **(Up)** and *A. mali* NBRC 102425 **(Down)**. In purple, *MarR* genes coding for antibiotic responsive transcriptional regulator; in blue, *MerR* gene coding for mercury resistance transcriptional regulator; in green, multidrug transporter genes; in red, multidrug permease genes; in orange, small multidrug resistance protein; in black, genes that are not involved in antibiotic resistance.

*Alicyclobacillus mali* FL18 genome was analyzed to identify putative heavy metal and/or antibiotic resistance genes. As already reported, these resistance genes may code for efflux pump able to confer resistance to both antibiotics and metals; these elements could have co-evolved or be genetically linked on transposable genetic elements (Knapp et al., 2017). From the *in silico* analysis of *A. mali* FL18, four genes encoding multidrug resistance (MDR) efflux transporters and five genes encoding putative transcriptional regulator of the MerR or MarR families (Figure 7 and Table 5) were detected. Genome analysis of *A. mali* NBRC 102425 revealed a higher number of multidrug transporters in comparison to *A. mali* FL18, suggesting that such different genetic profile is the result of adaptation to diverse environments. In *A. mali*, FL18 genes are distributed on five contigs and are not organized in canonical operon structures; only MarR transcriptional factor was found associated to putative MDR transporter in three contigs (k89_135, k89_146, and k89_147) (Figure 7).

**TABLE 5:** Annotation, strand, and length of the predicted heavy metal and/or antibiotic resistance genes.

Function	Strand	Length
Transcriptional regulator, MerR family	+	157
Transcriptional regulator, MarR family	−	136
Transcriptional regulator, MarR family	+	129
Transcriptional regulator, MarR family	−	135
Transcriptional regulator, MarR family	−	107
Multidrug-efflux transporter, major facilitator superfamily (MFS)	+	395
Multidrug-efflux transporter, major facilitator superfamily (MFS)	−	426
Multidrug-efflux transporter, major facilitator superfamily (MFS)	−	382
Permease, multidrug efflux	+	575
Small Multidrug resistance protein	+	344
Multidrug resistance transporter, Bcr/CflA family	+	1253
Undecaprenyl-diphosphatase BcrC	+	599

The MDR efflux transporters are widespread among microbes and recognize a variety of chemically and structurally different toxic compounds as well as antibiotics and are generally regulated by several families of transcription factors that modulate the expression in response to binding of drug molecules acting as effectors (Fiorentino et al., 2007; Contursi et al., 2013). These transcriptional regulators may belong to the MarR family, which is widespread across different bacterial species, probably due to horizontal gene transfer (Fiorentino et al., 2011; Beggs et al., 2020).

Noteworthy, *A. mali* FL18 contains in its genome a gene coding for a putative undecaprenyl-diphosphatase BcrC (NCBI Accession Number WP_067849704.1), which might be involved in the bacitracin resistance. In particular, BcrC in *Bacillus subtilis* is responsible for the dephosphorylation of UPP, an essential intermediate of the peptidoglycan biosynthesis. BcrC competes with bacitracin for UPP binding, promoting peptidoglycan biosynthesis, and its expression is activated by a broad range of conditions (antibiotics, ethanol, and salts) through a “damage-sensing” mechanism (Piepenbreier et al., 2020). To understand if this genetic trait was unique for *A. mali* FL18, the presence of BcrC in *A. mali* NBRC 102425 was also investigated. Surprisingly, the latter possesses the putative BcrC as well (NCBI Accession Number WP_067849704) and the two protein sequences share an identity of 100%, indicating that this gene is conserved within the species.

### Assessment of Arsenic Tolerance

To perform a phenotypic characterization regarding metal ion and antibiotic tolerance, the bacterial growth of *A. mali* FL18 was tested in different media. From the analysis of growth curves, the optimal broth was found to be BAT medium, in which the doubling time was 30 min (Supplementary Figure 1). Afterward, *A. mali* FL18 was cultivated in BAT medium and tested for its arsenic tolerance, showing MIC values of 11 mM and 41 mM for As(V) and As(III), respectively (Table 6). These findings indicate that *A. mali* FL18 is an arsenic-tolerant bacterium (Altowayti et al., 2020). The MIC value toward As(III) is comparable to that of other arsenic-tolerant microorganisms, ranging from about 10 to 50 mM (Das et al., 2014; Antonucci et al., 2017). Interestingly, the high MIC toward As(III) is close to that of two As(III) oxidizing strains of *Bacillus* and *Geobacillus* (16 and 47 mM, respectively) isolated from contaminated soils of West Bengal (Majumder et al., 2013).

**TABLE 6:** *Alicyclobacillus mali* FL18 tolerance to heavy metal ions.

MIC value	
As(V)	11 mM
As(III)	41 mM
Cd(II)	0.8 μM
Cu(II)	0.5 mM
Co(II)	3 mM
Ni(II)	30 mM
Hg(II)	17 μM

The observed As(III) tolerance can be explained by the occurrence of MDR transporters and by the presence of a higher number of genes specific for arsenite (arsenite methyltransferase, arsenite efflux systems, and organoarsenical permease) rather than arsenate resistance (arsenate reductase) (Figures 6, 7).

**FIGURE 8:**
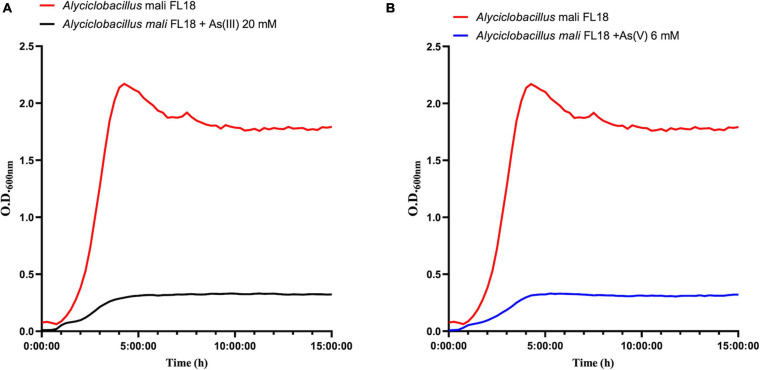
Growth curves of *A. mali* FL18 in the presence of 20 mM As(III) **(A)** and 6 mM As(V) **(B)**. The red curve is the As-free control growth.

To better compare the effect on cellular growth of As(V) and As(III), *A. mali* FL18 was cultivated at half of the MIC value (6 mM and 20 mM, respectively). As expected, a significant decrease in the duplication time was observed (Figure 8); indeed, the generation time was increased from 30 to 60 min in the presence of 20 mM As(III) and 6 mM As(V), respectively. The longer replication time can be traced back to arsenic toxicity, the effects of oxidative stress to the cells, and/or the time needed to activate the resistance pathways.

Furthermore, in order to verify if *A. mali* FL18 was able to perform arsenic bioconversion, a qualitative colorimetric assay was used to test the growth in the presence of arsenic ions.

**FIGURE 9:**
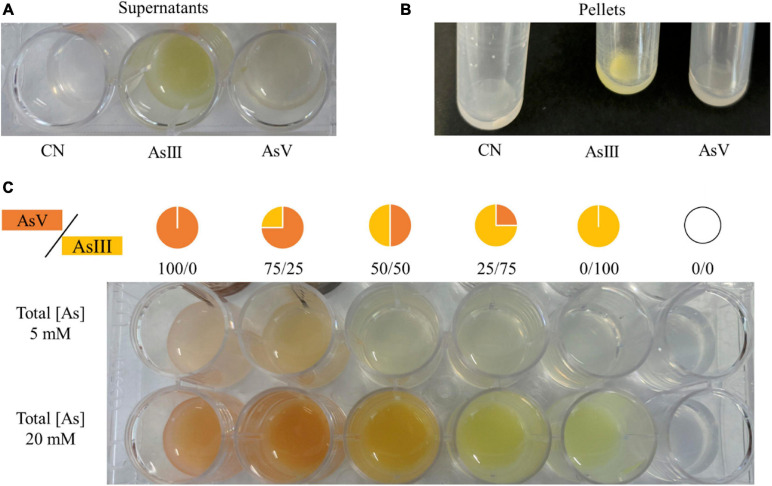
Qualitative evaluation of arsenic biotransformation performed by *A. mali* FL18. The experiment was performed by reaction of AgNO_3_ with **(A)** supernatants and **(B)** pellets of cells grown for 16 h in BAT medium (CN well) and in BAT medium supplemented with 20 mM As(III) or 5 mM As(V). **(C)** Color scales of different ratios of As(V)/As(III) in solutions with final arsenic concentrations of 5 and 20 mM.

When *A. mali* FL18 was cultivated in the presence of 20 mM As(III), a bright yellow precipitate was obtained in both supernatants and cells, suggesting that As(III) is not converted to As(V). Conversely, when *A. mali* FL18 was grown in the presence of 5 mM As(V), a color change was observed (Figure 9). Although quantitative and/or biochemical data are required, this experiment indicates that *A. mali* FL18 reduces As(V) and does not oxidize As(III), compatible with our genomic analysis, which highlighted the presence of a putative arsenate reductase but not of an arsenite oxidase (Table 4).

### Heavy Metals Tolerance in *A. mali* FL18

Solfataric environments are characterized by the coexistence of various toxic substances; in particular, at Pisciarelli, high levels of CO_2_, H_2_S, and NH_4_ coexist with As, Hg, Fe, Be, Ni, Co, and Cu (Aiuppa et al., 2006; Cardellini et al., 2017; Piochi et al., 2019). Table 6 shows that *A. mali* FL18 has a heterogeneous profile of metal tolerance; in particular, it exhibits high MIC values for nickel, cobalt, and mercury (30 mM, 3 mM, and 17 μM, respectively, Table 6) (Sharma and Shukla, 2020), but low for copper (0.5 mM). Regarding cadmium, the MIC value (0.8 μM) is indicative of an almost null tolerance, in agreement with the fact that the genome does not apparently contain any cadmium resistance gene and supporting the idea that, differently from other thermophilic microorganisms, the arsenic resistance system is specific and selective (Antonucci et al., 2018). Notably, Cd is virtually absent at Pisciarelli (Piochi et al., 2019).

### Antibiotic Susceptibility in *A. mali* FL18

Since the correlation between heavy metals and antibiotics is a well-known complex interaction (Ye et al., 2017) and due to the presence in the genome of genes coding for antibiotic resistance, we also analyzed the resistance of *A. mali* FL18 to antibiotics, determining the MIC values (Table 7).

**TABLE 7:** *Alicyclobacillus mali* FL18 susceptibility to antibiotics.

MIC value	
Ampicillin	20 μg/ml
Bacitracin	700 μg/ml
Chloramphenicol	<0.5 μg/ml
Ciprofloxacin	>1 mg/ml
Erythromycin	70 μg/ml
Kanamycin	80 μg/ml
Streptomycin	70 μg/ml
Tetracycline	<0.5 μg/ml
Vancomycin	1 μg/ml

With the aim to investigate susceptibility to antibiotics presenting diverse mechanisms of action, antibiotics belonging to different classes were chosen, particularly aminoglycosides (kanamycin and streptomycin), chloramphenicol, macrolides (erythromycin), and tetracyclines that inhibit protein synthesis; fluoroquinolones (ciprofloxacin) that inhibit the DNA replication; and β-lactams (ampicillin), glycopeptides (vancomycin), and antibiotic polypeptides (bacitracin) that inhibit the peptidoglycan synthesis in Gram-positive bacteria (Kapoor et al., 2017).

Interestingly, *A. mali* FL18 became resistant to all the antibiotics tested, except to chloramphenicol and tetracycline. The MIC values determined against ampicillin, erythromycin, kanamycin, streptomycin, and vancomycin were comparable to those of other antibiotic-resistant bacteria, as well as those relative to bacitracin (700 μg/ml) and ciprofloxacin (up to 1 mg/ml) (D’Aimmo et al., 2007; Minarini et al., 2012; Bulut et al., 2020). Besides the putative gene coding for BcrC and possibly responsible for bacitracin resistance (see above), it can be hypothesized that the expression of several multidrug-efflux transporters (Table 5) is responsible for the resistance to the other tested antibiotics. To the best of our knowledge, the co-occurrence of heavy metals and antibiotic resistance genes is a well-known mechanism in multidrug-resistant microorganisms such as *Pseudomonas aeruginosa* and *E. coli* (Nguyen et al., 2019), but not yet reported in extremophiles (Najar et al., 2020).

On the other hand, the resistance to ciprofloxacin is generally associated with mutations in the quinolone resistance-determining regions (QRDR) of *gyr*A and *par*C genes, respectively, coding for the subunit A of the DNA gyrase and the topoisomerase IV (Werner et al., 2010; Minarini et al., 2012). In particular, mutations in *gyr*A are able to confer resistance to ciprofloxacin, while mutations in *par*C can further improve the resistance (Hamouda and Amyes, 2004). Interestingly, in *A. mali* FL18, the subunit A of the DNA gyrase has two amino acid substitutions in correspondence to the QRDR, known to be responsible for the acquired resistance in *Enterobacteriaceae*, including *E. coli* (Minarini et al., 2012) (Supplementary Figure 2).

## Conclusion

Microbial bioremediation of toxic metals from the environment is a hot topic in the field of clean-up of contaminated sites; in this context, the exploitation of microbes flourishing in primordial niches rich in such toxic metalloids are very good candidates since they have been subjected to natural selection for a long time.

In this work, we identified and characterized a novel thermo-acidophilic bacterium, *A. mali* FL18, from a geothermal environment naturally rich in arsenic. Genome mining performed to target genes involved in metal detoxification revealed the presence of signatures typical of arsenic-tolerant microbes and of many multidrug efflux systems suggesting its ability to tolerate different toxic compounds.

Interestingly, the comparison of the repertoire of metal/drug resistance genes of our isolate with that of *A. mali* NBRC 102425 deposited on NCBI showed a higher number of arsenic resistance genes in *A. mali* FL18, mirroring a better potential in arsenic tolerance and suggesting that their different genetic profile is the result of their adaptation to specific niches. Analyses performed to test the capability of *A. mali* FL18 to tolerate heavy metals and different antibiotics confirmed its tolerance to a wide variety of different toxic compounds.

Altogether, these molecular and phenotypical features indicate that *A. mali* FL18 represents a suitable platform for further investigation either to address bioremediation of heavy-metal pollution and to identify new thermostable proteins/enzymes for industrial applications.

## Data Availability Statement

The datasets generated for this study can be found in online repositories. The names of the repository/repositories and accession number(s) can be found in the article/Supplementary Material.

## Author Contributions

MA, GG, and RP: data curation. MA, GG, RP, MP, AM, SB, and GF: investigation. MA, RP, GG, MP, AM, and GF: methodology. DL, PC, MP, SB, and GF: supervision. MA, GG, RP, MP, AM, SB, and GF: writing—review and editing. All authors have read and agreed to the published version of the manuscript.

## Conflict of Interest

The authors declare that the research was conducted in the absence of any commercial or financial relationships that could be construed as a potential conflict of interest.

## Footnotes

1 https://www.ebi.ac.uk/ena

2 https://orthovenn2.bioinfotoolkits.net/

3 https://www.ncbi.nlm.nih.gov/genome

4 https://www.ezbiocloud.net/tools/ani

5 http://ggdc.dsmz.de/ggdc.php#

## Abbreviations

As(V): arsenate
As(III): arsenite
Sb(III): antimony
Cd(II): cadmium
Cu(II): copper
Co(II): cobalt
Ni(II): nickel
Hg(II): mercury
MIC: Minimal Inhibitory Concentration
IARC: International Agency of Research on Cancer
UPP: undecaprenyl-pyrophosphate.
