# Supplementary material for: Genomic Insight of Alicyclobacillus mali FL18 Isolated From an Arsenic-Rich Hot Spring
Source: Front Microbiol. 2021 Apr 8;12:639697. doi: 10.3389/fmicb.2021.639697 (PMC8060452; doi:10.3389/fmicb.2021.639697)
Supplement: Supplementary file 1 [file Data_Sheet_1.docx]

| **COG number** | **COG description** |
| --- | --- |
| COG0012 | Predicted GTPase, probable translation factor [Translation, ribosomal structure and biogenesis]. |
| COG0013 | Alanyl-tRNA synthetase [Translation, ribosomal structure and biogenesis]. |
| COG0016 | Phenylalanyl-tRNA synthetase alpha subunit [Translation, ribosomal structure and biogenesis]. |
| COG0018 | Arginyl-tRNA synthetase [Translation, ribosomal structure and biogenesis]. |
| COG0030 | Dimethyladenosine transferase (rRNA methylation) [Translation, ribosomal structure and biogenesis]. |
| COG0041 | Phosphoribosylcarboxyaminoimidazole (NCAIR) mutase [Nucleotide transport and metabolism]. |
| COG0046 | Phosphoribosylformylglycinamidine (FGAM) synthase, synthetase domain [Nucleotide transport and metabolism]. |
| COG0048 | Ribosomal protein S12 [Translation, ribosomal structure and biogenesis]. |
| COG0049 | Ribosomal protein S7 [Translation, ribosomal structure and biogenesis]. |
| COG0051 | Ribosomal protein S10 [Translation, ribosomal structure and biogenesis]. |
| COG0052 | Ribosomal protein S2 [Translation, ribosomal structure and biogenesis]. |
| COG0072 | Phenylalanyl-tRNA synthetase beta subunit [Translation, ribosomal structure and biogenesis]. |
| COG0080 | Ribosomal protein L11 [Translation, ribosomal structure and biogenesis]. |
| COG0081 | Ribosomal protein L1 [Translation, ribosomal structure and biogenesis]. |
| COG0082 | Chorismate synthase [Amino acid transport and metabolism]. |
| COG0086 | DNA-directed RNA polymerase, beta' subunit/160 kD subunit [Transcription]. |
| COG0087 | Ribosomal protein L3 [Translation, ribosomal structure and biogenesis]. |
| COG0088 | Ribosomal protein L4 [Translation, ribosomal structure and biogenesis]. |
| COG0089 | Ribosomal protein L23 [Translation, ribosomal structure and biogenesis]. |
| COG0090 | Ribosomal protein L2 [Translation, ribosomal structure and biogenesis]. |
| COG0091 | Ribosomal protein L22 [Translation, ribosomal structure and biogenesis]. |
| COG0092 | Ribosomal protein S3 [Translation, ribosomal structure and biogenesis]. |
| COG0093 | Ribosomal protein L14 [Translation, ribosomal structure and biogenesis]. |
| COG0094 | Ribosomal protein L5 [Translation, ribosomal structure and biogenesis]. |
| COG0096 | Ribosomal protein S8 [Translation, ribosomal structure and biogenesis]. |
| COG0097 | Ribosomal protein L6P/L9E [Translation, ribosomal structure and biogenesis]. |
| COG0098 | Ribosomal protein S5 [Translation, ribosomal structure and biogenesis]. |
| COG0099 | Ribosomal protein S13 [Translation, ribosomal structure and biogenesis]. |
| COG0100 | Ribosomal protein S11 [Translation, ribosomal structure and biogenesis]. |
| COG0102 | Ribosomal protein L13 [Translation, ribosomal structure and biogenesis]. |
| COG0103 | Ribosomal protein S9 [Translation, ribosomal structure and biogenesis]. |
| COG0105 | Nucleoside diphosphate kinase [Nucleotide transport and metabolism]. |
| COG0126 | 3-phosphoglycerate kinase [Carbohydrate transport and metabolism]. |
| COG0127 | Xanthosine triphosphate pyrophosphatase [Nucleotide transport and metabolism]. |
| COG0130 | Pseudouridine synthase [Translation, ribosomal structure and biogenesis]. |
| COG0150 | Phosphoribosylaminoimidazole (AIR) synthetase [Nucleotide transport and metabolism]. |
| COG0151 | Phosphoribosylamine-glycine ligase [Nucleotide transport and metabolism]. |
| COG0164 | Ribonuclease HII [DNA replication, recombination, and repair]. |
| COG0172 | Seryl-tRNA synthetase [Translation, ribosomal structure and biogenesis]. |
| COG0185 | Ribosomal protein S19 [Translation, ribosomal structure and biogenesis]. |
| COG0186 | Ribosomal protein S17 [Translation, ribosomal structure and biogenesis]. |
| COG0215 | Cysteinyl-tRNA synthetase [Translation, ribosomal structure and biogenesis]. |
| COG0244 | Ribosomal protein L10 [Translation, ribosomal structure and biogenesis]. |
| COG0256 | Ribosomal protein L18 [Translation, ribosomal structure and biogenesis]. |
| COG0343 | Queuine/archaeosine tRNA-ribosyltransferase [Translation, ribosomal structure and biogenesis]. |
| COG0504 | CTP synthase (UTP-ammonia lyase) [Nucleotide transport and metabolism]. |
| COG0519 | GMP synthase, PP-ATPase domain/subunit [Nucleotide transport and metabolism]. |
| COG0532 | Translation initiation factor 2 (IF-2; GTPase) [Translation, ribosomal structure and biogenesis]. |
| COG0533 | Metal-dependent proteases with possible chaperone activity [Posttranslational modification, protein turnover, chaperones]. |

**Supplementary Table 1.** COG categories

| **OrthoANIu value (%)** | **98.91%** |
| --- | --- |
| **DDH estimate (GLM-based):** | **89.70% [87.4 - 91.7%]** |
| **Probability that DDH > 70% (i.e., same species):** | **95.7% (via logistic regression)** |
| **Probability that DDH > 79% (i.e., same subspecies):** | **64.81% (via logistic regression)** |

**﻿**

**Supplementary Table 2. OrthoANI and DDH values estimated from the comparison of *Alicyclobacillus mali* FL18 and NBRC 102425 genomes.**

| **Genome** | ***Alicyclobacillus mali* FL18** | ***Alicyclobacillus mali* NBRC 102425** |
| --- | --- | --- |
| **Domain** | **Bacteria** | **Bacteria** |
| **Size (bp)** | **3,024,307** | **2,786,970** |
| **GC Content (%)** | **61.5** | **61.9** |
| **N50 (bp)** | **124285** | **96664** |
| **L50** | **9** | **10** |
| **Number of Contigs (with PEGs)** | **48** | **85** |
| **Number of Subsystems** | **271** | **267** |
| **Number of Coding Sequences** | **3127** | **2815** |
| **Number of RNAs** | **64** | **63** |

**﻿**

**Supplementary Table 3.** Comparison between genome statistics of *A. mali* FL18 and NBRC 102425.


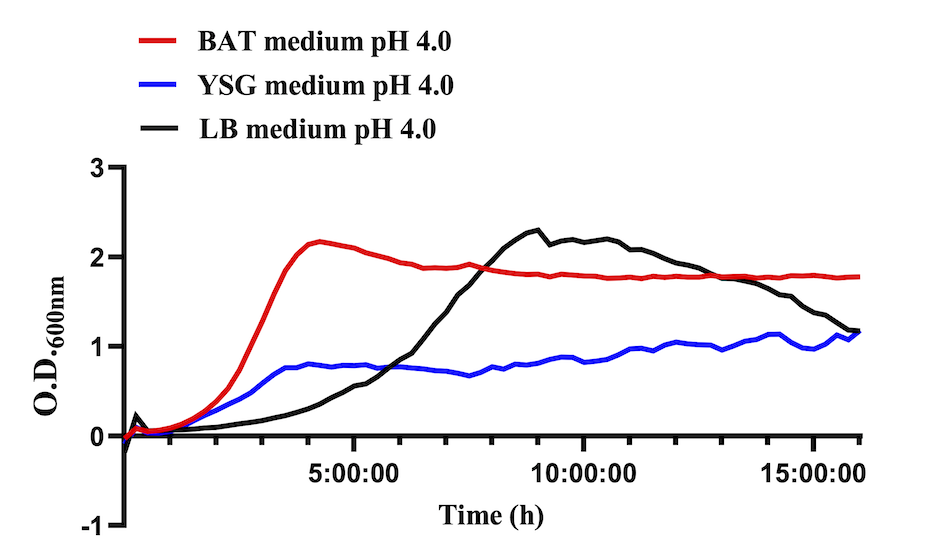


**Supplementary Fig. 1: Assessment of the *Alicyclobacillus mali* FL18 growth in different media.** The red line indicates the growth in BAT medium, the blue line indicates the growth in YSG medium and the black line the growth in LB medium.


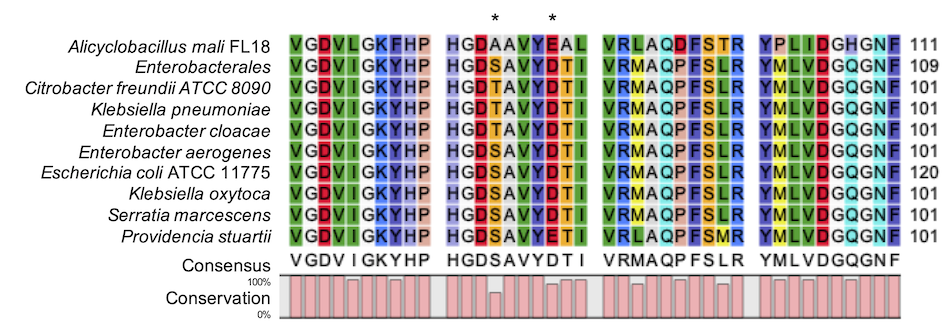


**Supplementary Fig. 2: Multiple Alignment of the DNA girase A of *Alicyclobacillus mali* FL18.** The aminoacidic mutations conferring resistance to ciprofloxacin are highlighted with an asterisk (*****). The NCBI accession numbers are reported in order of appearance: WP_195866958.1; WP_063586815.1; AAC68569.1; AAC68574.1; AAC68572.1; AAC68571.1; AAC68570.1; AAC68573.1; AAC68576.1; AAC68575.1.
